# Managing the Oocyte Meiotic Arrest—Lessons from Frogs and Jellyfish

**DOI:** 10.3390/cells9051150

**Published:** 2020-05-07

**Authors:** Catherine Jessus, Catriona Munro, Evelyn Houliston

**Affiliations:** 1 Laboratoire de Biologie du Développement - Institut de Biologie Paris Seine, LBD - IBPS, Sorbonne Université, CNRS, F-75005 Paris, France; 2 Laboratoire de Biologie du Développement de Villefranche-sur-mer (LBDV), Sorbonne Université, CNRS, 06230 Villefranche-sur-mer, France; catriona.munro@college-de-france.fr; 3 Inserm, Center for Interdisciplinary Research in Biology, Collège de France, PSL Research University, CNRS, 75005 Paris, France

**Keywords:** oocyte, meiosis, oogenesis, *Clytia*, *Xenopus*, maturation promoting factor (MPF), meiotic maturation

## Abstract

During oocyte development, meiosis arrests in prophase of the first division for a remarkably prolonged period firstly during oocyte growth, and then when awaiting the appropriate hormonal signals for egg release. This prophase arrest is finally unlocked when locally produced maturation initiation hormones (MIHs) trigger entry into M-phase. Here, we assess the current knowledge of the successive cellular and molecular mechanisms responsible for keeping meiotic progression on hold. We focus on two model organisms, the amphibian *Xenopus laevis*, and the hydrozoan jellyfish *Clytia hemisphaerica.* Conserved mechanisms govern the initial meiotic programme of the oocyte prior to oocyte growth and also, much later, the onset of mitotic divisions, via activation of two key kinase systems: Cdk1-Cyclin B/Gwl (MPF) for M-phase activation and Mos-MAPkinase to orchestrate polar body formation and cytostatic (CSF) arrest. In contrast, maintenance of the prophase state of the fully-grown oocyte is assured by highly specific mechanisms, reflecting enormous variation between species in MIHs, MIH receptors and their immediate downstream signalling response. Convergence of multiple signalling pathway components to promote MPF activation in some oocytes, including *Xenopus*, is likely a heritage of the complex evolutionary history of spawning regulation, but also helps ensure a robust and reliable mechanism for gamete production.

## 1. Introduction

Successful embryonic development following fertilisation in all animals critically depends on the quality of the gametes. In the case of the female gamete, the egg, this requires that two essential aspects of oogenesis are carefully controlled and coordinated: the meiotic divisions responsible for producing a maternal haploid genome, and the accumulation of maternal mRNAs, proteins and organelle stockpiles required to support early embryonic development. A key feature of oogenesis is that the meiotic divisions arrest during prophase of the first division, this arrest being held while oocyte growth is completed and then until conditions are optimal for egg release. This chapter focuses on the successive mechanisms responsible for maintaining this oocyte meiotic prophase I arrest.

Oogenesis is initiated when oogonia, the female reproductive stem cells, complete premeiotic S-phase and enter the first meiotic division. Now referred to as “oocytes”, they undergo the four first stages of meiotic prophase: leptotene, zygotene, pachytene and diplotene. During these stages, chromosome pairing, crossover formation and recombination between homologous parental chromosomes are achieved. Then, the first meiotic division arrests for prolonged periods at the diplotene stage, which depending on species can last days, weeks, months or years. During this long-lasting arrest, the oocytes grow enormously, accumulating all the molecular reserves required for the early embryonic development. Stockpiled maternal mRNAs and proteins originate from the oocytes own transcriptional activity, but also by transfer from closely associated nurse and/or follicle cells. Accumulation of yolk proteins rich in lipids and carbohydrates (vitellogenesis) involves both their synthesis by the oocyte and endocytic uptake from surrounding fluids following production in diverse organs such as the vertebrate liver, mollusc oocyte follicle/auxiliary cells or the insect fat body [1,2,3,4]. Oocyte growth is complete when transcription and vitellogenesis stop. At this point, the fully-grown oocytes become ready to undergo the process of oocyte maturation, which accompanies ovulation or spawning. Oocyte maturation crucially involves the resumption of meiosis, as well as species-specific organisational changes that, for instance, prepare the egg for fertilisation. This key process is described in detail in the following sections. In almost all species, oocyte maturation is triggered by “Maturation Initiation Hormones” (MIHs), secreted molecules with a wide variety of molecular natures that are delivered by surrounding cells. A notable exception concerns mammals, where cGMP exchange between the follicle cells and the oocyte through gap junctions fulfils the same signalling role [5]. For the rest of this chapter, we will expand the term “MIH” to cover this trans-cellular cyclic nucleotide signalling, as well as the secreted hormones used more commonly.

The delivery of these species-specific MIH signals is regulated by upstream hormonal and environmental inputs, which have evolved in order to optimise the moment of egg release for successful fertilisation. In vertebrates, for example, hormonal readouts of stress and nutritional balance converge to regulate the production of gonadotropins along the Hypothalamic–Pituitary–Gonadal axis, culminating with release of Luteinising Hormone (LH) from the pituitary to stimulate the oocyte follicle cells [6]. Local signals from these follicle cells (or equivalent cells in other animals) then act on the oocyte to trigger meiotic maturation.

From this overview, it is apparent that at least three types of mechanism maintain the prophase state of the oocyte during its long life. First there is a programmed pause in germ cell development between synapsis and chromosome segregation, whose nature is largely unknown. Then, during oocyte growth, safeguards against premature meiotic maturation avoid the production of haploid and fertilisable oocytes of insufficient size. Finally, fully-grown oocytes develop a responsiveness to specialised MIH signals that initiates the maturation process only when the physiological and environmental conditions are favourable.

### 1.1. The “Universal” Prophase Arrest

Oocytes derive from Primordial germ cells (PGCs), which colonise and divide in the developing gonad according to species-specific developmental programs. In many species, they are channelled into the germ cell program, and, more specifically, the oogenesis program in females, in response to cues from somatic gonad tissues [7,8,9,10]. Oogonia (ovary reproductive stem cells) then undergo additional proliferative divisions and a pre-meiotic S-phase followed by entry into the highly specialised prophase of the first meiotic division, which is extended to allow parental chromosome pairing, crossing-over and recombination.

As a general rule, these early stages of oogenesis are completed in the developing gonad before adulthood. Thus, most oocytes in the adult ovary are characterised by an arrest at the prophase of the first meiotic division. Nevertheless, small populations of mitotically active oogonial stem cells can be detected in the adult ovaries in many species, including *Drosophila*, fish, amphibians, mouse and human [11,12,13,14].

It is during the period of prophase I arrest, which may last many years in some animals, that oocyte growth occurs. The cellular processes responsible for this massive cellular expansion are multiple and vary between species. As presented in more detail in the following sections, these include RNA and protein synthesis from the oocyte genome, endogenous mitochondrial replication, uptake of yolk (vitellus) from blood or other surrounding fluids and direct supply of materials from nurse cells/follicle cells. The presence of four genome copies in the prophase I oocyte not only facilitates massive RNA and protein synthesis, but also minimises the impact of potentially deleterious haploid mutations and protects the genome by allowing accurate DNA repair using highly active homologous recombination-based mechanisms [15].

Once oocyte growth is completed, the prophase block is ready to be released by physiological triggers for oocyte maturation and ovulation. A loose chromatin configuration indicates that most growing oocytes are held at the diplotene stage. In some species, including *Xenopus* and *Clytia*, marked chromosome condensation in the largest ovarian oocytes can be observed. The greater chromosome condensation observed in some species may reflect progress through prophase into the diakinesis stage. In *Xenopus,* diakinesis is fully achieved only once oocyte meiotic maturation is underway [16]. After oocyte maturation and ovulation are completed, emptying the ovary of all fully-grown oocytes, a new population of smaller oocytes will complete growth for ovulation in the following cycle. The number of oocytes that grow to full size during each reproductive cycle depends on a variety of factors, such as nutrient availability and season/temperature. Thus, adult gonads contain mixtures of different oocyte growth stages, but all these are arrested at meiotic prophase I.

**Figure 1:**
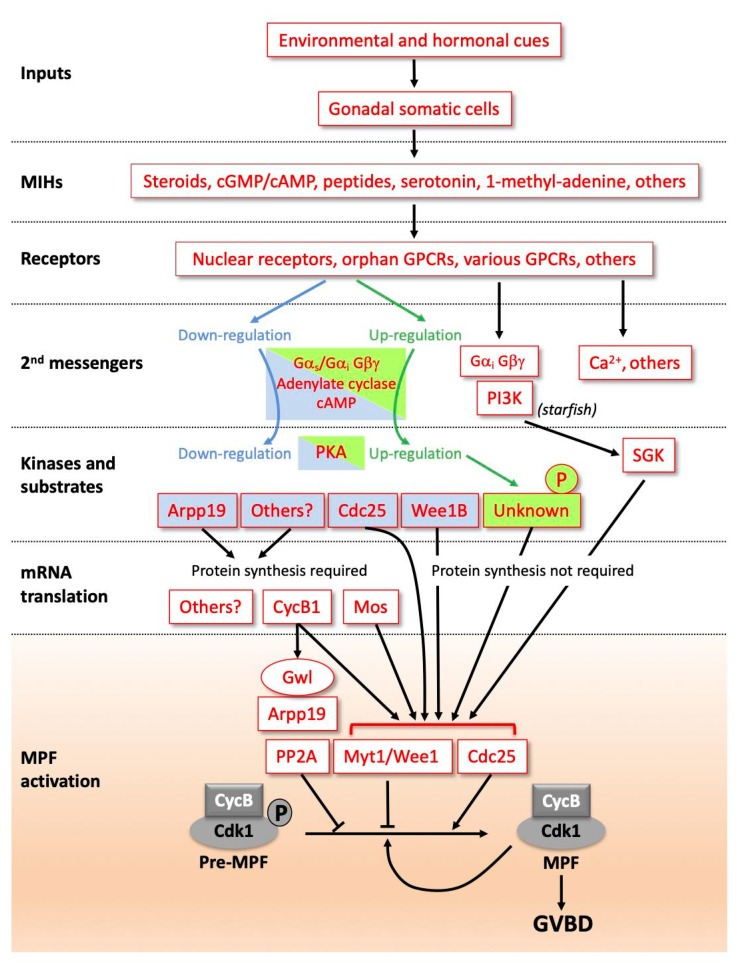
Diverse signalling pathways initiated by maturation initiation hormones (MIHs) lead to a universal biochemical core, activating maturation promoting factor (MPF). Due to the diversity of molecular pathways controlling the resumption of meiosis, this scheme is not exhaustive but is based on a few examples among those studied. MIHs are released from somatic cells near the oocyte (follicle cells, ectoderm cells or others) in response to hormonal (luteinizing hormone (LH) in vertebrates or gonad-stimulating substance (GSS) in starfish) and environmental inputs (dark/light transition, temperature, sea water, etc.). MIH molecules have different identities between species, and the types of receptors mediating the MIH action are also diverse; see text for details. In amphibian and fish oocytes, steroids recruit canonical nuclear receptors unusually associated with membranes in oocytes, but also progestin-specific membrane receptors most probably acting through Gα_i_ and Gβγ. MIH receptor activation leads either to the downregulation of adenylate cyclase, a decrease in cAMP level and PKA activity in vertebrates, or the opposite regulation in various species of hydrozoans or nemerteans. In vertebrates, PKA substrates dephosphorylated following the drop in PKA activity include Arpp19 in *Xenopus*, Cdc25 in *Xenopus* and mouse, and Wee1B in mouse. PKA substrates remain to be identified in species where PKA activity increases. In starfish, the dissociation of Gα_i_ from Gβγ activates phosphoinositide 3-kinase (PI3K) which leads to serum- and glucocorticoid-regulated kinase (SGK) activation, independently of cAMP and PKA. In other species, cytoplasmic calcium release is critical in the pathway. In many vertebrate oocytes, the drop in PKA activity indirectly activates the synthesis of new proteins required for MPF activation, such as Cyclin B1 and, in *Xenopus,* the kinase Mos. In many other species, MPF is activated without the need for new synthesized proteins. The final step of MPF activation (orange box) is common to all animals. Cyclin B-Cdk1 is activated by Cdk1 dephosphorylation at T14 and Y15 due to the reverse of the balance of activities between its regulators, the phosphatase Cdc25 and the kinases Wee1/Myt1. This activation is accelerated by an auto-amplification loop. In parallel, the Cdk1 opposing enzyme, the PP2A phosphatase, is inhibited by Arpp19 phosphorylated by the kinase Greatwall (Gwl).

Whilst the initial arrest of female germ cells during first meiotic prophase seems to be a conserved aspect of oocyte development and thus can be considered “universal”, the same is not true for the mechanisms that hold the fully-grown oocytes in prophase until they receive the MIH signal (Figure 1). As mentioned above, MIH molecules have widely different molecular identities between species, and this is reflected in the types of receptors to which they bind, and to the signalling pathways activated in immediate response to MIH-receptor binding. There are also differences in the events that follow. The two meiotic divisions may be fully completed during oocyte maturation or they may show secondary arrests in the body cavity and/or awaiting the sperm, for instance at metaphase I in ascidians or metaphase II in vertebrates [17]. Despite these differences, the core biochemical events that mediate entry into the first meiotic M-phase following MIH stimulation are remarkably constant in all animals. Of key importance is a kinase–phosphatase system centering on “auto-amplification” of the maturation promoting factor (MPF; now rebranded as M-phase promoting factor), a molecular complex of the kinase Cdk1 and its regulatory subunit Cyclin B. Because of its key importance in oocyte maturation, the molecular regulation of MPF auto-amplification during oocyte maturation is described in detail in the following section. Another conserved biochemical pathway leads to the cytoplasmic accumulation during oocyte maturation of a second factor called cytostatic factor (CSF), responsible for the “secondary” arrest of oocytes awaiting fertilisation. Whether the meiotic cycle arrest is in metaphase I, metaphase II or whether a complete cycle ends with a G1 arrest, it is the Mos-MEK-MAPkinase-p90^Rsk^ kinase cascade that provides the CSF activity responsible for this arrest in all species studied to date, targeting different proteins according to the stage of the cycle arrest [18,19,20,21,22].

### 1.2. From MIH Stimulation to MPF Activation

Studies using amphibian and starfish oocytes contributed to the identification and eventual molecular characterisation of the two key oocyte maturation factors described above, MPF and CSF [21,23,24,25,26,27,28]. Subsequent studies have led to a good understanding of the biochemical regulation of maturation in several fish species [29] and in mammals, especially mouse [5]. Additional knowledge has come from other diverse species, including *Caenorhabditis elegans* [30], *Drosophila* [31], *Cerebratulus* [32], molluscs and annelids [1]. From these, we can present a global scheme in which signalling pathways initiated by MIH lead to the activation of the MPF. The main steps are outlined in Figure 1. MIH is released from somatic cells near the oocyte in response to hormonal and environmental inputs. Examples of molecules that can act as MIHs include steroids in amphibian and fish [29,33], 1-methyl-adenine in starfish [34], serotonin in bivalve molluscs and nemertean worms [1,32,35,36], and short amidated peptides in hydrozoans [37]. Reflecting this molecular diversity of MIHs, their receptors on the oocyte surface appear to be of several distinct types, and to act via different second messenger systems. Nevertheless, as detailed below, it is striking that receptors of the G-protein coupled receptor (GPCR) class, which signal via dissociation of associated heterotrimeric G proteins into Gα and Gβγ subunits, have been implicated in the oocyte maturation response in many animals, either as MIH receptors, as counter-balancing regulators, and/or as receptors for upstream hormones [38].

The oocyte’s immediate response to MIH-receptor binding in all vertebrates is a decrease in oocyte cAMP levels caused by the down-regulation of adenylate cyclase. As a result, the activity of cAMP-dependent kinase (PKA) in the oocyte cytoplasm drops. The modulation of cAMP concentration and PKA activity downstream of MIH is also critical for meiotic resumption in many non-vertebrate species, including hydrozoans such as *Clytia*, but in these species they act as positive rather than negative regulators [39]. In other species, distinct signalling systems are deployed, for example Gβγ and phosphoinositide 3-kinase (PI3K) in starfish [40,41,42]. Much remains to be understood about the pathways that connect these various second messenger systems to MPF activation, which, even between vertebrate species, show considerable variation. *Xenopus* is currently one of the best understood cases, with several PKA substrates demonstrated to participate in the MIH response (Figure 1). One important component of the oocyte prophase lock in this context is the small protein Arpp19 [43]. Dephosphorylation of Arpp19 at the PKA site around serine 109 (S109) following MIH stimulation of the oocyte is a necessary step in the pathway leading to MPF activation [43]. Dephosphorylation of a PKA site on the MPF-activating phosphatase Cdc25 is also important for this transition in both mouse and *Xenopus* oocytes [44,45,46,47], while in mouse oocytes an equivalent PKA regulation additionally suppresses activity of the MPF inhibitory kinase Wee1B [48]. Yet another essential event linking PKA downregulation to MPF activation in the majority of vertebrates, although not in small rodents, is new protein synthesis from mRNAs stockpiled into the cytoplasm. In *Xenopus*, two critical de novo synthesized proteins contribute to MPF activation, Cyclin B1 and the kinase Mos [49]. Cyclin B1 synthesis is the key to triggering MPF auto-amplification. Cyclin B1 binds to monomeric Cdk1, forming an active MPF complex that phosphorylates and so activates its own direct regulators, the inhibitory kinases Myt1 and Wee1, and the activatory phosphatase Cdc25. In parallel, Cyclin B1 synthesis sets off another pathway that enhances the effects of MPF by inhibiting the phosphatase PP2A, whose activity is widely responsible for counterbalancing that of Cdk1 through the cell cycle. PP2A inhibition is achieved via a second role for Arpp19, which at the time of MPF activation becomes phosphorylated on a distinct site (Serine 67) by the kinase Greatwall (Gwl) [33,50]. In parallel, newly-synthesized Mos in MIH-stimulated oocytes activates MEK and thereby MAPkinase, a pathway that has conserved oocyte roles in CSF arrest as mentioned above, and also in orchestrating the unequal divisions necessary for polar body formation [18,51,52,53]. In *Xenopus,* but not other species examined, this pathway also contributes to the regulation of Myt1/Wee1 and Cdc25 [33]. In various species of invertebrates and in a few vertebrates, including small rodents, the synthesis of new proteins is not required for MPF activation, which in these species occurs much more quickly following MIH stimulation.

The final step of the MIH-induced signalling pathways is identical and universal in oocytes of all animal species: Cyclin B-Cdk1 activation is initiated by any trigger that reverses the balance of activities between Cdc25 and Wee1/Myt1. It is then further accelerated by the auto-amplification process, as described above. This MPF activation is coupled with the parallel inhibition of its opposing enzyme, the PP2A phosphatase, by the Arpp19-Greatwall duo.

### 1.3. Overview of Oogenesis and Oocyte Maturation in Clytia and in Xenopus

**Figure 2:**
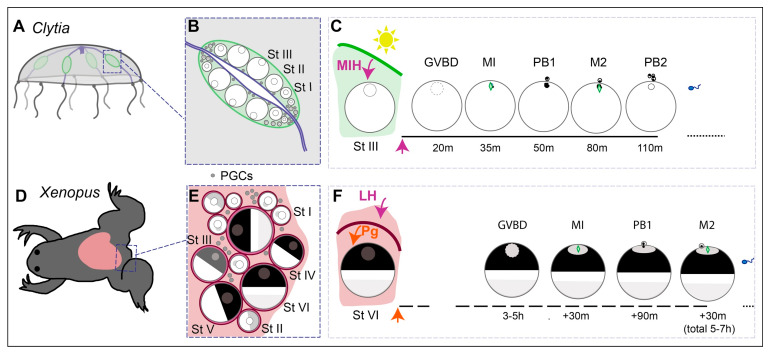
Overview of oocyte growth and maturation in *Clytia* and in *Xenopus.* (**A**) In *Clytia* adult jellyfish, oocytes develop throughout adult life in the four gonads (green), situated on each of the radial gastrovascular canals connected to the central feeding organ (blue). (**B**) Stage I, II and III growing oocytes are sandwiched between an outer ectoderm and the endodermal layer of the central gastric cavity, which directly provides nutrients for growth. (**C**) A dark–light transition each dawn causes MIH to be released from specialised cells of the gonad ectoderm. MIH acts on an oocyte GPCR leading to MPF activation, manifest as Germinal Vesicle Breakdown (GVBD), then completion of meiotic divisions MI and M2, with the emission of polar bodies PB1 and PB2, before spawning as a G1 unfertilised egg after about two hours. (**D**) In the adult *Xenopus* ovary (pink), the meiotic cycle of oocytes is also arrested at diplotene of the first meiotic prophase. (**E**) Growing oocytes of Stages I-VI are tightly surrounded by one layer of follicle cells and a theca (blood vessels, collagen, fibroblasts) (magenta). (**F**) Ovulation is triggered by Luteinising hormone (LH) from the pituitary, which causes follicle cells to release steroid hormones, including progesterone (Pg). These act on oocyte membrane receptors to initiate a series of events culminating with variable timing in GVBD and completion of the first meiotic division before arrest as an unfertilised egg at M2 after a total of around 5–7 h.

To illustrate conserved and variable features of oogenesis and oocyte maturation regulation in relation to the maintenance of the meiotic prophase I block, we will first provide an overview of the current understanding of these processes in amphibians (especially *Xenopus laevis*) as a long-standing vertebrate model, and in the emerging cnidarian model *Clytia hemisphaerica*, as schematised in Figure 2.

In *Clytia*, as in other hydrozoan cnidarians, germ cells originate from a multipotent stem cell population called i-cells (interstitial cells) that express conserved germ cell/stem cell gene families such as *Nanos* and *Piwi* [54]. These reside in the anastomosing stolon system that connects the feeding and budding polyps of the benthic “colony” form from which the jellyfish emerge. Growth of newly budded *Clytia* jellyfish to sexual maturity is completed in 2–3 weeks in laboratory conditions. In female jellyfish, definitive germ cells first can be recognised within the first week of growth following budding. As described in detail in the following section, oocyte growth proceeds through two main phases. The first occurs close to the i-cell pools in peripheral regions of the gonad, while the second, major, phase follows their repositioning to the flanks of the gonad and tight association with the nutritive endoderm cell layer [55,56].

Oocyte maturation in hydrozoans including *Clytia*, followed by the release of the unfertilised egg through the ectoderm of the gonad into the surrounding seawater, occurs in response to a dark–light transition at dawn (and/or the reverse transition at dusk in some species). The same cues promote the activation and release of sperm from the gonads of males, thus allowing gametes to be released simultaneously into the seawater when the *Clytia* jellyfish gather at the ocean surface at dawn [37]. They act by triggering the secretion of *Clytia* MIH from light-sensitive cells in the gonad ectoderm that contain an essential Opsin protein [57]. *Clytia* MIH is a short amidated peptide with the sequence W/P-R-P-A/Y/Pamide [37]. From phylogenetic analysis of its receptor, it can be placed in a family of neuropeptide hormones that regulate reproductive processes across the animal kingdom. The oocyte MIH receptor, MIHR, is a GPCR that signals via Gα_S_ and an immediate early rise in ooplasmic cAMP [38,58]. The subsequent activation of PKA leads by an unknown mechanism to the activation of MPF, manifest as the breakdown of the large oocyte nucleus (GVBD for Germinal Vesicle Breakdown) within 10–15 min. The two meiotic divisions are completed within two hours. At this point, the egg is released through the gonad ectoderm, and held at G1 of the first mitotic cycle by a Mos-MAPkinase-dependent CSF arrest until fertilisation [18].

In amphibians, oocytes originate from PGCs that migrate into the genital ridges, the first rudiments of the gonads, during embryogenesis. Oogenesis starts when the gonad undergoes sexual differentiation during metamorphosis, and continues in the ovary throughout adulthood. Proliferating oogonia complete premeiotic S-phase, followed by the chromosome pairing and recombination events of prophase of the first meiotic division. The subsequent arrest at the diplotene stage lasts for 2.5 to 3 years in *Xenopus*. During this very long arrest, the oocyte grows within a follicle comprising one layer of tightly associated cells and surrounded by a theca (see Section 2 for details). As in *Clytia,* oocyte transcription and vitellogenesis turn off at the end of the growth period. Only then does the fully-grown oocyte become responsive to MIH to trigger meiotic maturation. In amphibians, the essential component of MIH is the steroid hormone progesterone, delivered by the surrounding follicle cells in response to the ovulatory gonadotropin LH. Oocyte maturation takes 6–8 h, producing an unfertilised egg arrested by CSF at meiotic metaphase II.

### 1.4. Prophase Arrest Mechanisms Acting in Two Main Phases

Although the prophase arrest of ovarian oocytes is often described as “universal”, its prolonged maintenance does not rely on a single underlying mechanism. Rather, we can consider that distinct mechanisms act in successive phases. First, molecular checkpoints found in all meiotic cells delay entry into M-phase until chromosome pairing and meiotic recombination have occurred. These include a specific “pachytene checkpoint” (also known as the meiotic recombination checkpoint) that arrests or delays chromosome separation if recombination is not complete [59], and also the canonical DNA damage checkpoint [60]. An additional specific feature of the oocyte development programme is that the diplotene to diakinesis transition is generally put on hold as cell growth commences. Little is known about the underlying mechanisms for this, and so they will not be discussed further here, but they may involve elements of these two checkpoints. Subsequently, during the long period of oocyte growth, premature meiosis resumption is prevented until the oocyte is sufficiently stocked with molecular reserves to support successful embryonic development. As the components responsible for the maturation response to hormonal signalling are progressively set in place, they must not be allowed to trigger premature meiotic maturation. As discussed in detail in Section 2 for the amphibian case, this can be achieved by leaving the synthesis of key components until the last stages of growth. Finally, at the end of the growth period, distinct, species-specific, mechanisms to prevent M-phase entry come into play that are integral parts of the switches that allow fully-grown oocytes to respond to MIH signals. Additional hormonal systems relating to oocyte growth but also to environmental or physiological status can prevent the MIH response. These mechanisms are discussed in Section 3.

## 2. A Pause in Meiosis to Allow Oocyte Growth

Before discussing how prophase arrest is maintained during oocyte growth in *Clytia* and *Xenopus*, we will describe what is known of the cellular processes that mediate this highly specialised process of cell growth.

### 2.1. Massive Cell Growth Combining Different Modes

#### 2.1.1. Xenopus

**Figure 3:**
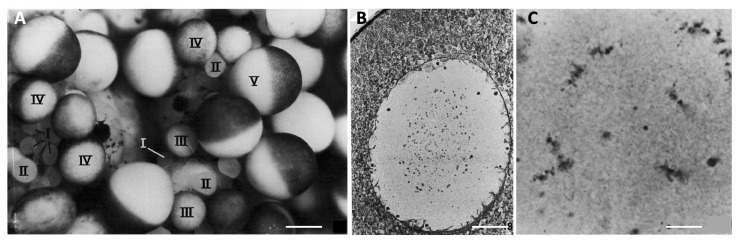
Oocyte growth in *Xenopus laevis.* (**A**) A portion of the ovary from a recently ovulated female. Oocytes at successive stages of growth are indicated by Roman numerals. Note the absence of fully-grown Stage VI oocytes (characterised by a completely unpigmented equatorial band), which were ovulated. Scale bar 0.5 mm. (**B**) Micrograph of a paraffin section of a Stage V oocyte, hematoxylin and eosin staining. The animal pole of the oocyte is at the top left of the micrograph. Chromosomes and small nucleoli condense at the center of the nucleus. Scale bar 14 μm. (**C**) Micrograph of a paraffin section of a Stage VI oocyte showing the condensed lampbrush chomosomes. Scale bar 1.8 μm. The images were reproduced with permission (license number 4818271464898) from J.N. Dumont, Journal of Morphology; published by John Wiley and Sons, 1972 [61].

Each ovary of the adult female *Xenopus* forms a bag comprising a hilum and about 20 lobed pouches containing oocytes at all stages of growth. Oocyte growth has been divided into six stages, based mainly on oocyte size and the distribution of yolk and pigment [61] (Figure 3A). Oocyte transcriptional activity is uncoupled from the uptake of nutrients from the thecal blood vessels: transcription occurs during Stages I to IV, including first the production of mRNAs involved in directing embryonic development (Stages I and II), and then ribosomal RNAs (Stages III and IV), while vitellogenesis takes place mainly from Stage III to Stage V, peaking during Stage IV. Stage I oocytes, 50 to 200 μm in diameter, are transparent, with the nucleus occupying a large part at the centre of the cell. They are covered by a thin layer of follicle cells surrounded by the theca, a layer containing collagen, fibrocytes and blood vessels. Early during Stage I, oocytes transition from pachytene to early diplotene. During Stage II (200 to 400 μm), the oocyte becomes opaque as some yolk appears. The follicle cells make close contact and develop gap junctions with the oocyte, which develops microvilli [62,63]. At this stage, an acellular vitelline envelope forms around the oocyte. At Stage III (400 to 600 μm), the uptake and accumulation of yolk are initiated in parallel with transcription of ribosomal RNA from highly extended loops of decondensed chromatin, termed lampbrushes, notably stockpiling ribosomes for use during embryogenesis. The transcription of vitellogenin genes is induced in the liver by estrogen; subsequent transport of the yolk protein in the blood and its specific uptake by the oocyte are under combined regulation by estrogen and gonadotropins [64]. Another hormonal regulator of oocyte growth in *Xenopus* is insulin, or more likely the related insulin like-growth factor-1 (IGF-1) whose receptor transcripts reach the highest levels when glycogen synthase activity is maximum [65]. Both accelerate oocyte growth in vitro [66], while IGF-1, more efficiently than insulin, enhances the transport of blood glucose across the oocyte membrane [67].

During Stage IV (600 to 900 μm) the characteristic animal–vegetal polarity of the oocyte develops, with the dark brown pigment that started to accumulate earlier becoming strongly segregated to the animal cortex. The vegetal hemisphere becomes filled with large yolk platelets formed by the fusion of vitellogenin-filled endocytic vesicles. The lampbrush chromosomes begin to retract and undergo condensation, marking the end of the oocyte transcriptional activity. At Stage V (900 μm to 1.1 mm), a distinct boundary forms between the two hemispheres, yolk uptake strongly decreases and the enlarged oocyte nucleus, termed the germinal vesicle (GV), is displaced towards the animal pole (Figure 3). Finally, in fully-grown, Stage VI, oocytes (1.1 to 1.3 mm), yolk uptake and transcription stop completely, microvilli retract and the chromosomes become shorter and more condensed (Figure 3).

The duration of each stage of oogenesis has not been precisely estimated, however, a new population of Stage VI oocytes can be restored in the ovary within one year of a loss of 70% through spawning. Early reports further suggested that proliferating oogonia in the ovaries of adult amphibians provide successive generations of oocytes [68,69,70]. This issue merits fresh investigation. An estimated stock of 240,000–250,000 diplotene oocytes present in the ovaries after the first year of adult life would, in any case, be sufficient to supply oocytes well beyond the active sexual life of the adult (about 20 years) [71].

#### 2.1.2. Clytia

**Figure 4:**
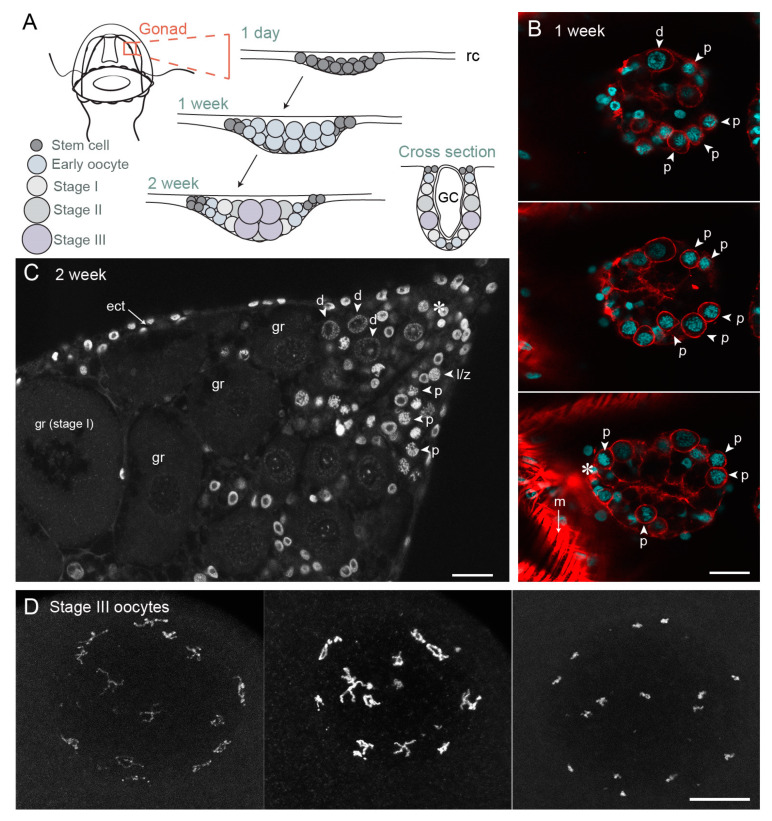
Gonad development and oocyte growth in *Clytia hemisphaerica*. (**A**) Schematic of gonad development in 1-day jellyfish (newly released from a polyp specialised for budding called the gonozooid), one-week and two-week old jellyfish; (**B**) Confocal image through the gonad of one-week old jellyfish, three different z planes; cyan—Hoechst (DNA), red—phalloidin (actin). In the bottom section, strong phalloidin staining of a surrounding fold of bell muscle is visible (m); (**C**) Gonad of two-week-old jellyfish, single confocal z plane. Grey—Hoechst (DNA). (**D**) Maximum projection of fully-grown Stage III oocytes at different stages of chromatin compaction. The fifteen pairs of homologous chromosomes, linked by chiasmata, are dispersed throughout the nucleus, mostly adjacent to the nuclear envelope. Grey—Hoechst (DNA). Asterix indicates possible stem cells. Arrows indicate different meiotic stages in early oocytes. Abbreviations: ect—ectoderm, GC—gastric cavity, rc—radial canal, gr—growing oocyte, l/z—leptotene/zygotene stage oocyte, p—pachytene stage oocyte, d—diplotene stage oocyte, m—muscle. Scale bars all 20 µm.

Within the gonads of *Clytia* jellyfish, oocytes or spermatogonia derive from cells that express orthologues of conserved germ line proteins including Nanos and Piwi (Figure 2 and Figure 4). Other Nanos/Piwi-expressing cells occupy sites at the base of the tentacles and of the central feeding organ [54]. All these Nanos/Piwi-expressing cells are generally equated with the characteristic multipotent hydrozoan stem cell type, the i-cells, but they also likely include fate-restricted precursors of somatic cells (neuroblasts, nematoblasts and gland cell precursors) or, in the case of the gonad, germ cells. A self-maintaining germ-cell stem-cell (GCSC) population has been shown to separate from the i-cell lineage in the sexual polyp forms of the hydrozoans *Hydra* and *Hydractinia* [72,73], but is yet to be demonstrated or characterised in *Clytia.* Unlike *Hydractina* and *Hydra, Clytia* has a “full” hydrozoan life cycle in which a pelagic jellyfish form buds from specialised polyps to assure sexual reproduction [74]. Sex is determined early during development of the polyp colonies from which the jellyfish buds [75], and is likely intrinsic to the i-cells [76], which, in *Clytia* reside in the anastomosing stolon system that connects the polyps.

After female jellyfish bud from the colony and start to feed and grow, small developing gonads first can be recognised within one week, as Nanos/Piwi-expressing cells positioned at the sites of gonad formation on the four radial canals embark on oogenesis. Germ line cells proliferate and grow at these sites, and the morphology of each gonad develops as a pouch hanging from beneath the bell. It has a simple organisation with two somatic epithelial layers, ectodermal and endodermal, sandwiching the developing germ cells [55,56]. After about one week of jellyfish growth, a first cohort of oocytes has entered meiosis and displays paired chromosomes with the characteristic configurations of pachytene (Figure 4).

Oocyte growth in *Clytia* occurs in two main phases. Initial growth starts in parallel with advancement through meiotic prophase I from pachytene to diplotene, indicating that synapsis is complete. The emerging gonads of one-week old jellyfish are largely composed of small oocytes at these stages, measuring between around 7–14 µm in diameter (Figure 4B). Oocytes of these early stages are also found in peripheral regions of female gonads throughout their adult life, which lasts about one month (Figure 4C). We hypothesize that these peripheral diplotene oocytes are unable to proceed to the major growth phase until they transit to the flanks of the ovary to establish contacts with the nutritive endoderm. Early diplotene oocytes (14–20 µm in diameter) undergo some growth prior to reaching the major growth phase (Figure 4C). Additional small oocytes may be generated throughout adult life from i-cells/GCSCs. Consistent with this possibility, functional gonads can reform following complete ablation [77], the gametes likely deriving from i-cell recruitment from pools at somatic sites.

During the major growth phase, three stages of oocyte growth have been defined [55,56]. Stage I oocytes (50–120 µm in diameter) are characterised by a single large nucleolus, and a GV positioned centrally within the oocyte. Stage II oocytes (120–160 µm in diameter) are characterised both by size and by the fragmentation of the nucleolus. Towards the end of Stage II the GV starts to reposition towards the future animal pole [55]. Stage III oocytes (160–180 µm in diameter) have no visible nucleolus and the GV is positioned peripherally at the animal pole. Shortly after spawning, Stage II and III oocytes are completely absent from the gonad, suggesting that some proportion of Stage I oocytes become committed to growth after daily spawning. Analysis of size distributions during the daily reproductive cycle indicates that growth from Stage I to Stage III takes about 13–15 h in laboratory culture conditions [55,56]. During this period, the oocyte chromatin remains in the partly-decondensed diplotene state (Figure 4C). At the end of the growth phase, chromosome recondensation is observed, likely over the course of several hours, and the individual chromosomes become highly compact (Figure 4D). These fully-grown oocytes are now responsive to MIH.

Growth of the *Clytia* oocyte from Stage I to Stage III likely involves both the continued synthesis of macromolecules and organelles by the oocyte itself and the transfer of material from the endoderm cells [56,78,79]. From Stage I onwards, yolk vesicles accumulate rapidly in the egg. As reported in other hydrozoans, this vitellogenesis appears to occur largely through synthesis within the oocyte from prominent Golgi systems, rather than by endocytotic/pinocytotic uptake of vesicles from surrounding fluids [78,79]. In parallel, the oocytes become tightly associated on their future vegetal pole side with cells of the nutritive endoderm cell layer, allowing accelerated cell growth. This endodermal cell layer is directly involved in the uptake of nutrients from the central gonad digestive cavity (see Figure 4A) by phagocytosis and absorption [77]. Basal projections of the endoderm cells are in close contact with the oocytes and cytoplasmic passages can be detected between the two [56]. The mode of material transfer from the endoderm cells to the oocytes, which appears to proceed in parallel to the endogenous synthesis of yolk vesicles by the oocyte Golgi, remains to be characterised in *Clytia*. Quite diverse oocyte growth mechanisms have been reported amongst different hydrozoan species. In some, materials are provided rather by i-cell-derived “nurse cells”, which, in the extreme case of *Hydra*, ends with apoptosis of the nurse cells and their phagocytosis by the oocytes [80,81,82].

Indirect evidence suggests that protein synthesis in the oocyte during growth is, in part, under the control of TOR/Akt signalling. As in many other growing cells, mRNAs translated during oocyte growth include mRNAs with specialised 5′cap structures called 5′TOP mRNAs whose translation is typically stimulated by TOR kinase via the activation of p70 ribosomal protein S6 kinase [83]. In oocytes of many animals, the TOR pathway is downstream of insulin–InsR signalling [84,85]. Amongst the proteins synthesized from 5′TOP mRNAs in growing *Clytia* oocytes is Mos2, one of the two *Clytia* Mos kinase paralogues expressed in the oocyte [18]. Morpholino injection experiments indicate that *Clytia* Mos2 has an atypical role in preparing the oocyte for maturation. In contrast, Mos1 is translated only after MIH stimulation and has retained a role, highly conserved across animal species, in regulating meiotic spindle positioning and also the post-meiotic “CSF” arrest of the oocyte.

These overviews of oogenesis in *Xenopus* and *Clytia* illustrate how growth occurs during the meiotic prophase block. The basic programmes of intense mRNA and protein synthesis are supplemented by additional cellular mechanisms that show species-specific differences. These include variable contributions of yolk uptake and yolk synthesis, as well as direct cytoplasm and organelle transfer from different types of neighbouring cells by phagocytosis and/or cytoplasmic bridges. The many differences in oocyte growth mechanisms likely reflect rapid and repeated evolutionary changes in egg size and seasonality under the influence of ever-changing environmental selective pressures and reproductive strategies. Although the contribution of neighbouring cells is variable, oocyte growth universally depends on communication between the germ line and the soma. A soma–germ-cell dialog assures uptake, storage and metabolic cooperation during the growth phase, involving local reciprocal interactions with nurse cells and follicle cells as well as long-distance communication. An example of this in vertebrates is vitellogenin production by the liver in response to estradiol from ovarian follicle cells [64]. In *Xenopus*, the oocyte’s growth stage dictates the response of the follicle cells to the gonadotropin LH [86]. Follicle cells surrounding growing oocytes produce estradiol, whereas those around Stage VI oocytes produce progesterone, as discussed in the next section.

The size reached by the oocytes and correspondingly the amount of nutrients made available to the future embryo is also highly variable according to species (1.3 mm in diameter in *Xenopus*, 180 μm in *Clytia*). It correlates partly with the duration of embryogenesis until feeding is possible. One other important difference between the two models is that the fully-grown oocyte remains quiescent for much longer in *Xenopus* than in *Clytia*, i.e., for several months rather than just a few hours each day. This may have important implications for the molecular mechanisms discussed in the following sections that firstly prevent growing oocytes prematurely exiting the prophase block in response to MIH, and then later prevent fully-grown oocytes from “leaky” responses at inappropriate times.

### 2.2. Progressive Acquisition of the Competence to Resume Meiosis

#### 2.2.1. Xenopus

The progressive shift during oocyte growth from an unresponsive to a responsive state regarding meiosis resumption is well-illustrated in amphibians. Although the meiotic cycle is arrested at diplotene of prophase I throughout oocyte growth, only large Stage V and fully-grown VI oocytes are responsive to progesterone. Most studies of this phenomenon have been based on comparisons between “small” Stage IV oocytes, which are unresponsive to progesterone, and competent Stage VI fully-grown oocytes. The inability of small oocytes to respond to progesterone can reflect deficiencies in any of the regulatory steps of meiotic maturation resumption outlined above (Figure 1), i.e.,: (i) the delivery of steroids, mainly progesterone, from follicle cells to the oocyte and the activation of membrane receptors; (ii) the rapid inhibition of adenylate cyclase leading to a drop in cytoplasmic cAMP and a subsequent decrease in PKA activity; (iii) the synthesis of critical proteins, notably the kinase Mos, indirectly responsible for MAPkinase activation, and Cyclin B1; (iv) activation and auto-amplification of MPF; (v) phosphorylation of the multiple targets of Cdk1 that organise the structural events of cell division, notably nuclear envelope breakdown and formation and functioning of the meiotic spindles. These various blocks coexist during early stages of oocyte growth, ensuring a strong lock in prophase. They progressively disappear as the oocyte grows, until all are released from Stage V onwards.

##### (i) Delivery of MIH by Follicle Cells

In the amphibian ovary, as in all vertebrates, the diplotene oocyte is surrounded by steroidogenic follicle cells (Figure 2). An LH surge promotes the release of the oocyte into the oviduct (ovulation) and simultaneously triggers the oocyte to advance from prophase I, ultimately arresting as an unfertilised egg in metaphase II. In amphibians, LH acts on the follicle cells surrounding Stage VI oocytes to stimulate a local release of steroids that directly trigger meiotic maturation [87,88,89]. Progesterone was initially proposed to be the steroid responsible for triggering oocyte meiotic maturation in *Rana pipiens* and *Xenopus* [87,90,91,92,93]. Testosterone was thereafter reported as the main steroid produced in response to LH [94,95,96], and induces in vitro meiotic maturation as efficiently as progesterone. Surprisingly, many other steroids are able to induce the maturation of isolated oocytes, such as pregnenolone, progesterone, RU486 (a progesterone receptor antagonist), dehydroepiandrosterone (DHEA), androstenedione, testosterone, digitoxigenin, brassinosteroids or cyclodextrin (for review: [33]). Interestingly, neither estradiol nor other estrogens induce meiotic maturation.

A study based on gas chromatography coupled to mass spectrometry allowed identification of steroids released by follicle cells at the time of ovulation [97]. The prophase-arrested oocyte contains low levels of testosterone and estrogens, whereas progesterone is almost undetectable. Progesterone is the first and the major steroid produced by follicle cells around Stage VI oocytes after LH stimulation, increasing abruptly to micromolar concentrations. Follicle cells surrounding growing oocytes rather produce estradiol, thereby promoting vitellogenesis [86]. These observations favour the view that progesterone plays a major physiological role in triggering meiotic maturation. Nevertheless, given the complexity of the follicular steroid landscape, it cannot be excluded that other steroids may be involved in the resumption of meiosis, in particular, testosterone.

Surprisingly, the oocyte has high steroid sulfate contents [97]. Sulfation transforms a liposoluble steroid into a water-soluble molecule, thereby abolishing some biological activities. The sulfotransferase activity in follicle cells acts as a buffering system, protecting the oocyte against local variations in active steroids that could trigger oocyte maturation at an inappropriate time. At the time of ovulation, the LH surge induces a strong production of progesterone that cannot be sulfated, hence triggering oocyte meiotic maturation. Sulfation by surrounding follicle cells also helps to protect small oocytes from a response to local steroids present outside the ovulation period.

##### (ii) Regulation of Oocyte cAMP Levels and the PKA Response

Several studies have found that adenylate cyclase activity becomes inhibited upon progesterone treatment in both Stage IV and Stage VI oocytes, resulting in both cases in a decrease in cAMP levels in the cytoplasm [98,99]. Stage IV oocytes thus already possess a steroid receptor able to downregulate cAMP levels and PKA activity. Indeed, the two receptors proposed to mediate progesterone action, the classical nuclear progesterone receptor and the membrane progestin receptor (see Section 3), are already expressed as proteins in Stage IV oocytes [100,101]. In smaller oocytes (Stage I to III), progesterone also inhibits adenylate cyclase, but less efficiently [102]. Hence, the coupling between progesterone and adenylate cyclase is not fully functional at the beginning of the growth period and is established when oocytes reach Stage IV. Furthermore, microinjection of PKI, a specific inhibitor of PKA which induces maturation in fully-grown oocytes [103,104], does not provoke MPF activation in Stage IV oocytes [99], showing that the response downstream of PKA is defective at this stage.

##### (iii) Translational Control

One of the events downstream of the cAMP step is the synthesis of new proteins required for MPF activation, most critically Mos and Cyclin B1 [28,33,105]. Stage IV oocytes fail to accumulate both proteins in response to progesterone [106], despite the presence of the mRNAs. The failure of Stage IV oocytes to initiate maturation in response to progesterone thus reflects their failure to mobilise Cyclin B and Mos mRNAs.

##### (iv) MPF Activation

Another piece of essential molecular machinery unable to function correctly in Stage IV oocytes is the last step of MPF activation. In fully-grown oocytes, the MPF complex is present as inactive “pre-MPF”. Cdk1 is maintained inactive by phosphorylations on T14 and Y15 maintained by the kinase Myt1 (Figure 1). Conversion of pre-MPF into MPF requires dephosphorylation of these sites by the phosphatase Cdc25, with the activation of Cdc25 and inactivation of Myt1 being reinforced by phosphorylative regulation by Cdk1 itself, in the so-called MPF “auto-amplification” mechanism. This auto-amplification is initiated by the formation of a small amount of active MPF, which can be generated by the binding of newly synthesized B-Cyclins to monomeric Cdk1 [107,108]. Pre-MPF as well as Cdc25 and Myt1 are present in incompetent Stage IV oocytes [106,109,110]. Although nuclear envelope breakdown (GVBD) can be triggered in these growing oocytes by microinjection of egg cytoplasm containing MPF activity [99,111,112], Y15 dephosphorylation of endogenous Cdk1 is not complete [113]. Moreover, when B-Cyclins are injected into Stage IV oocytes, they associate with endogenous Cdk1, but the ectopic complexes are immediately inactivated by phosphorylation on Y15 [109]. These small oocytes are thus unable to generate active new complexes that could trigger the auto-amplification mechanism. From these results, we can conclude that MPF activation is locked during oogenesis at the level of both the generation of the active MPF trigger and the positive feedback loop between Cdk1 and Cdc25/Myt1. Another of the crucial limiting factors accounting for the incompetence of small oocytes when re-entering meiosis in response to progesterone is Plx1 kinase, a central player in the MPF auto-amplification loop, which is not expressed in Stage IV oocytes [106].

##### (v) MPF Targets

If MPF activity is generated artificially in Stage IV oocytes, by cytoplasmic transfer from matured oocytes [93,103,105], or by injecting Cyclin B and Plx1 simultaneously [106], or by injecting Cyclins together with an inhibitor of the phosphatase PP2A [109,111], which is the enzyme antagonizing MPF activity [114], the cellular events of the first meiotic division do not proceed normally. GVBD occurs, but the nuclear contents do not rise to the animal pole of the cell, as they do in large oocytes to form the characteristic “white spot” of maturing oocytes [99,109,111]. Moreover, although chromosomes undergo some condensation, they remain spread throughout the cytoplasm and no metaphase spindle forms [109,111]. These observations indicate that, at Stage IV, some of the targets of MPF are absent or inaccessible, preventing the normal progression of the meiotic divisions.

To summarise, the prophase arrest of growing Xenopus oocytes is assured by multiple factors preventing the oocyte to resume meiotic divisions in response to local hormonal signals. The signalling pathway leading from progesterone stimulation to adenylate cyclase activation is established by Stage IV, but at that time the MPF amplification system cannot not be primed by Cyclin B synthesis, or activated due to lack of Plx1, while MPF substrates are also missing or masked. As the oocyte grows further, these missing elements are restored progressively, so that the Stage VI oocyte can respond fully to meiosis induction signals.

#### 2.2.2. Clytia

In *Clytia* adult female gonads, following each daily spawning, cohorts of Stage I oocytes embark on growth and progress to Stage III. In laboratory culture conditions, this rapid growth is completed within around 15–16 h, as shown by the ability of the oocytes to mature when treated with cell-permeable cAMP analogues [56]. Competence to mature in response to the physiological dark–light cue shows similar timing, as reported in other hydrozoan species [58]. Maturation competence is thus acquired within a much shorter timeframe than in *Xenopus*, with premature maturation a risk for only about 14 h for each oocyte during its main growth phase. Almost nothing is known about how the machinery governing the different events of meiotic resumption is set up during oocyte growth in *Clytia*, so the following section is necessarily speculative. We can nevertheless again consider how deficiencies in each step of the normal maturation response could potentially prevent premature maturation until growth is complete. In the case of *Clytia*, these successive events are: (i) delivery of MIH peptides from gonad ectoderm neural cells to the oocyte and the activation of MIHR on the oocyte surface; (ii) Gα_s_-mediated activation of adenylate cyclase leading to a rapid rise in cytoplasmic cAMP and PKA activation; (iii) possible synthesis of unknown protein(s); (iv) activation and auto-amplification of MPF; (v) phosphorylation of Cdk1 targets for GVBD and entry into MI. As we have seen during *Xenopus* oocyte growth, it is likely that the machinery responsible for each of these five steps is put in place partly in parallel.

##### (i) MIH Delivery and Receptor Binding

The stores of MIH neuropeptide vesicles in the specialised gonad ectoderm neuroendocrine cells become considerably depleted each morning when MIH is released, but these stores are largely replenished within an hour [37]. MIH availability is thus unlikely to limit premature maturation of growing oocytes. It remains possible, however, that the Opsin9-dependent machinery that allows these cells to release MIH shows a period of reduced responsiveness. The GPCR responsible for mediating the maturation response, MIHR (see below), is synthesized from an mRNA detectable already in growing (Stage I-II) oocytes, but at 3- 4 times higher high levels in fully-grown (Stage III) oocytes [38]. Regulation of the timing of translation of these mRNAs and/or of insertion of the protein into the plasma membrane could potentially be important safeguards of the prophase block during oocyte growth. In the scallop gonad, increasing expression of serotonin receptors on the oocyte membrane during oocyte growth is under the control of estradiol [1,115].

##### (ii) MIHR Signal Transduction Machinery

No information is available about when the cytoplasmic signal transduction components: Gα_S_, adenylate cyclase, cAMP and PKA become functionally linked to the MIHR during oocyte growth. mRNAs for all these components are present in growing (Stage I-II) and fully-grown (Stage III) *Clytia* oocytes (unpublished analyses of transcriptomes [37] available for searching at http://marimba.obs-vlfr.fr/). Potential candidate for keeping the growing oocyte unresponsive to MIH are the PKA substrate(s) responsible for linking the initial MIH signalling to MPF activation, whose identities remain an important gap in our knowledge. A highly conserved ortholog of Arpp19, the key PKA substrate for *Xenopus* maturation initiation, is expressed in *Clytia* oocytes. Given the opposite roles of PKA in these two species, it will be of interest to test whether this acts as a PKA substrate during the response to MIH, and, if so, how its interacting partners compare with those of PKA-phosphorylated *Xenopus* Arpp19.

##### (iii) Protein Synthesis

As reported in many other animals, a Mos family MAPkinase kinase kinase, *Clytia* Mos1, becomes rapidly translated by the oocyte following MIH stimulation and cAMP signalling [18]. Its activity is vital for correct polar body formation by regulating the cortical positioning of the two meiotic spindles. It is also required for the cell cycle arrest of the unfertilised egg at G1 of the first mitotic cycle, once maturation is complete. Unlike in *Xenopus* oocytes, however, Mos1 translation does not impact MPF activation, but rather the two kinase cascades appear to operate in parallel. GVBD can occur as quickly as 10 min after treatment with MIH, suggesting that all the necessary protein components for MPF activation are already present, as seen also in echinoderm oocytes [21]. The marked increase in chromosome condensation observed in Stage III oocytes (Figure 4D) suggests that MPF initial steps activation may occur ahead of the MIH trigger, potentially reflecting the MIH response system taking over to hold the prophase arrest. Nevertheless, our unpublished results show that cAMP-induced GVBD is sensitive to pretreatment with the translation inhibitor emetine, suggesting the involvement of the neosynthesis of one of more proteins very early in maturation induction. Similar results were obtained using the TOR inhibitor rapamycin, indicating that this synthesis may be under TOR regulation.

##### (iv, v) MPF Activation and Phosphorylation of Its Downstream Targets

As with signal transduction machinery components, mRNAs for the main molecular actors of MPF auto-amplification (Cdk1, Cyclin B, Cdc25, Wee1) are present in growing and fully-grown oocyte transcriptomes. Given the rapidity of GVBD, it is highly likely that entry into the first meiotic M-phase, as well as the assembly and function of the first meiotic spindle, is carried essentially by existing proteins.

To summarise, the issue of how growing oocytes remain refractile to dark–light transitions until they are fully grown remains to be addressed experimentally in *Clytia*. Given that the major oocyte growth phase is completed in less than a day, the danger of the premature ovulation of undersized eggs is relatively low. Existing knowledge suggests synthesis of MIHR and/or its insertion into the plasma membrane may occur relatively late during growth, and/or that cAMP downstream components are limiting. It would not be surprising if, as in *Xenopus*, more than one contributing factor helps to lock the oocyte in prophase until growth is complete.

## 3. The Second Arrest: The Oocyte Poised for Maturation

We have seen that in fully-grown oocytes the machinery to respond to MIH is fully assembled. The oocyte is poised to mature once the appropriate hormonal and environmental conditions are signalled. Maintenance of the prophase block at this stage thus depends on the active maintenance of a signal transduction pathway in the “off” state. These signalling pathways vary widely between different species, as exemplified by the comparison between *Clytia* and *Xenopus*.

In *Xenopus* as in other vertebrates, cytoplasmic cAMP concentrations, and consequently the activity of PKA, are maintained high in the arrested oocyte and must be down-regulated to promote meiotic resumption. In contrast, the elevation of cAMP levels and PKA activity are required to promote release from the prophase block in many species including hydrozoans such as *Clytia,* as well as in phylogenetically scattered species including nemerteans and some (but not all) annelids, molluscs, ascidians, and echinoderms [39,58]. In this context, we outline here recent advances in *Xenopus* and *Clytia* in the understanding of the oocyte receptors that respond to MIH stimuli upstream of cAMP, and of the PKA substrates that link this to MPF activation.

### 3.1. Xenopus

**Figure 5:**
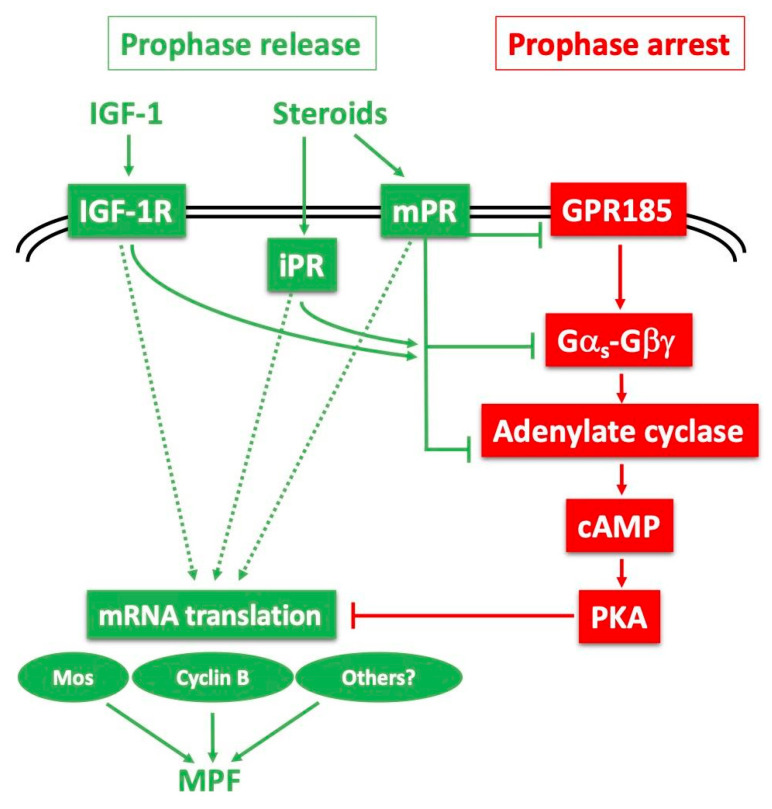
Models for release of the *Xenopus* oocyte prophase block: probable cooperation between several receptors and downstream pathways. The constitutively active Gα_s_-coupled GPR185 maintains high cAMP and PKA activity, ensuring the prophase arrest (red pathway—right). The prophase release (green pathway—left) is triggered by steroids, mainly progesterone, with the potential contribution of IGF-1 and its receptor (IGF-1R). Progesterone could interact with its canonical nuclear receptor (iPR) but also a plasma membrane-bound receptor (mPR). Upon binding to progesterone, these receptors could inhibit the GPR185 pathway, and/or independently inhibit adenylate cyclase by recruiting Gα_i_ or inhibiting Gα_S_. They could also launch positive downstream signalling independently of cAMP and PKA. These cascades converge to the synthesis of new proteins required for activating MPF.

The identity and molecular mechanism of the *Xenopus* oocyte MIH receptor is still not resolved. Here, we will present the evidence that several distinct receptors, and probably more than one associated signalling pathway, participate in maintaining the prophase arrest of the fully-grown oocyte (summarised in Figure 5). The prophase arrest of the *Xenopus* oocyte is intimately linked to maintenance of high levels of cAMP by a constitutively active GPCR, GPR185 (formerly GRPx) [116,117]. *Xenopus* GRP185 is closely related to the orphan G-protein coupled receptors GPR3 (mouse) and GPR12 (rat), that also act as essential mediators of meiotic arrest via Gα_S_ and its stimulation of adenylate cyclase [118,119,120]. Heterotrimeric G protein activity associated with *Xenopus* GPR185 may involve Gβγ as well as Gα_S_ activating the cAMP signalling pathway [121,122,123,124,125]. The down-regulation of GPR185 activity following the stimulation of follicles by gonadotropin involves protease-mediated cleavage of the receptor [116], and/or progesterone-induced endocytosis [126], consistently with the importance of vesicle trafficking in maintaining meiotic arrest [127,128]. Inactivation of this receptor is not, however, sufficient to trigger the resumption of meiosis [116,117].

The initiation of oocyte maturation requires the direct activation of a distinct receptor for the steroid MIH, whose identification has been an ongoing challenge for more than half a century. Such a receptor is expected not to show the properties of a conventional steroid receptor. It should interact with membranes, since progesterone acts at the level of surface or internal membranes [101,129,130,131,132]. It is also expected to show low specificity towards steroids (the steroid moiety of cardiac glycosides, digitoxigenin [133], or plant brassinosteroids [33], induce *Xenopus* oocyte maturation), and to act via the modulation of adenylate cyclase activity. Since 2000, several candidates have been proposed as oocyte receptors for progesterone or testosterone. The first was the conventional transcriptional progesterone receptor present within the oocyte cytoplasm, which can be partly recovered in membrane fractions and is proposed to act in an unconventional, non-genomic manner in the oocyte [100,134,135,136,137,138]. Similarly, testosterone may elicit a non-genomic response in the *Xenopus* oocyte via a classical androgen receptor, also partly associated with membranes [96,136,139]. It has further been proposed that cross-talk between classical testosterone and progesterone receptors is able to attenuate GPR185 signalling [140]. The discovery of a new family of membrane progestin receptors (mPRs), unrelated to nuclear steroid receptors, but instead having some of the characteristics of G protein-coupled receptors, has provided a plausible alternative mechanistic explanation of how progesterone acting at the cell surface of the oocyte can cause rapid intracellular responses independent of GRP185 [101,141]. To summarise, it is probable that a panel of steroids, mainly progesterone and testosterone, switch on both classical and membrane-associated steroid receptors to inactivate GPR185 and/or directly inactivate Gα_S_ or recruit Gα_i_, as well as possibly to act via cAMP-independent targets, to trigger meiotic maturation (Figure 5).

In addition to steroids, insulin and IGF-1 can induce *Xenopus* oocyte maturation in vitro [142,143,144] through the activation of the *Xenopus* IGF-1 receptor [145,146,147]. The IGF-1 receptor protein can be detected at the surface of fully-grown oocytes and during meiotic maturation [147]. The product of the proto-oncogene Ras, a well-established mediator of insulin action in somatic cells, is also able to induce the meiotic maturation of *Xenopus* oocytes [148,149], but is not necessary for the resumption of meiosis induced by either insulin/IGF-1 or progesterone [150]. It is now becoming evident that progesterone and insulin/IGF-1 share common intermediates to lead to Cdk1 activation, notably the cAMP drop and the requirement for protein synthesis, including Mos [149,151,152]. The in vivo physiological relevance of IGF-1 in meiotic maturation is not yet understood, but it could be to amplify the steroid action on meiosis resumption (Figure 5). In mammals, an intra-ovarian IGF system including receptors and binding proteins amplifies the action of gonadotropin on the follicles by increasing granulosa cell proliferation and steroidogenesis [153].

Regardless of which receptors mediate the response to MIH steroids, there is a general consensus that the key early consequence is a drop in cAMP levels in the oocyte. Many studies have shown that progesterone causes, within a few minutes, a modest decrease (20%) in the concentration of cAMP [154] resulting from the inhibition of adenylate cyclase activity [155,156,157,158] present as different isoforms in the oocyte [124,159]. In contrast, progesterone has no effect in vivo on the opposite enzyme, phosphodiesterase [151]. Furthermore, the inhibition of adenylate cyclase is GTP-dependent, and the activation of Gα_S_ either by cholera toxin or by overexpression inhibits progesterone-induced oocyte maturation [125,131,160], while loss of Gα_S_ function causes spontaneous meiotic resumption [121,125]. Similarly, the overexpression of Gβγ inhibits progesterone-dependent oocyte maturation [96,122,123], whereas its inhibition initiates spontaneous maturation [123] or enhances progesterone-induced oocyte maturation [122]. In the oocytes of some fish species, the MIH activity of progestins has been shown to be mediated directly by membrane receptors (mPRα and mPRβ) that signal through Gα_i_, a Gα subunit that inhibits rather than activates adenylate cyclase activity [141]. The involvement of such receptor-Gα_i_ signalling in *Xenopus* oocytes appears unlikely, since the specific inhibitor, pertussis toxin, does not block meiotic maturation [161,162,163]. The relationship between mPRs and classic GPCRs is discussed further in Section 5.

The main target of cAMP is the regulatory subunit of the cAMP-dependent protein kinase, PKA. PKA exists as a holoenzyme consisting of two regulatory subunits and two catalytic subunits. Upon the binding of cAMP to the regulatory subunits, the two catalytic subunits are released and become active [164]. The basal cAMP concentration in *Xenopus* prophase oocytes is close to the apparent activation constant of type II protein kinase A for cAMP [160,165], which is the predominant form of PKA in *Xenopus* oocytes [166,167]. Therefore, a modest reduction in oocyte cAMP by progesterone has functionally significant effects on PKA activity. It has been shown that progesterone causes an almost complete loss of PKA activity within 30 min, long before MAPkinase and MPF activation that is detected only several hours after progesterone addition [168,169]. PKA inhibition is thereafter maintained during meiotic maturation and is truly an early event, dissociable from all other biochemical (activation of numerous protein kinases, including MPF and MAPkinase) and cytological (GVBD) events that are dependent on de novo protein synthesis [168,169,170].

Substantial evidence indicates that the decrease in PKA activity is essential for the release of the prophase block. Notably, increased PKA activity by injection of its catalytic subunit blocks the ability of progesterone to initiate oocyte maturation [103]. Conversely, the inhibition of PKA activity by microinjection of either the regulatory R subunit of PKA or the specific inhibitor of PKA, PKI, leads to the re-initiation of oocyte maturation in the absence of progesterone [103,104]. Despite this general consensus that a decrease in cAMP and PKA activity is necessary and sufficient for meiosis re-initiation, one research group has proposed that progesterone triggers meiotic maturation independently of Gα_S_-adenylate cyclase-cAMP-PKA, bypassing the negative inhibitory signal imposed by the cAMP-PKA via an independent positive signal [171]. This hypothesis is difficult to reconcile with the evidence presented above, accrued from more than 40 years of research in many laboratories. It is compatible, however, with the possibility, mentioned above, that multiple receptors are likely involved in the control of meiosis, one (GPR185) for maintaining the prophase arrest, the other(s) for triggering meiotic resumption, possibly acting through both cAMP-dependent and independent pathways (Figure 5). Whether progesterone inactivates GRP185 in addition to activating positive maturation initiation receptors remains open.

As already outlined in Section 2, several PKA substrates have been identified in amphibian oocytes that contribute to linking MIH stimulation to downstream MPF activation. The first to be characterised was the dual-specificity phosphatase Cdc25, which phosphorylates and activates Cdk1. In the prophase-arrested oocyte, Cdc25 is phosphorylated at S287 by PKA, which inhibits the ability of Cdc25 to dephosphorylate and activate Cdk1 [44]. It seems unlikely, however, that Cdc25 dephosphorylation is the critical early switch that unlocks the signalling pathway leading to MPF activation. Dephosphorylation of Cdc25 at S287 is only detectable several hours after PKA inhibition, at the time of MPF activation, and furthermore this dephosphorylation depends on Cdk1 activity [44]. Another substrate of PKA is the kinase Wee1, which opposes Cdc25 action by inhibitory phosphorylation of Cdk1 on amino acid residues T14 and Y15. In mouse prophase oocytes, Wee1 phosphorylation by PKA enhances its inhibitory effect on meiosis resumption [48]. However, Wee1 is not present in *Xenopus* oocytes, where an alternative T14/Y15 kinase, Myt1, holds Cdk1 inactive. Wee1 only becomes expressed later, after completion of the first meiotic division [172], so cannot contribute to the prophase arrest of *Xenopus* oocytes.

A very important PKA substrate for maintaining the *Xenopus* oocyte prophase arrest is Arpp19. Arpp19 and its alternatively spliced variant Arpp16 (Arpp for cAMP-regulated phospho-protein) were originally identified as proteins phosphorylated by PKA upon dopaminergic stimulation in the striatum [173,174]. Arpp19 is ubiquitously expressed in neurons and non-neuronal cells, whereas Arpp16 is enriched in neostriatum [175]. Various cellular functions have been attributed to the PKA phosphorylation of these proteins, although none are related to cell division [174]. The properties of Arpp19 match those of a heat- and acid-stable 20kDa protein isolated 30 years earlier from *Xenopus* prophase-arrested oocytes, described as a PKA substrate and dephosphorylated one hour after progesterone stimulation [176]. This similarity prompted analysis of Arpp19 in *Xenopus* oocytes and its identification as a PKA substrate that maintains the prophase arrest [43]. Arpp19 is phosphorylated by PKA at S109, a serine residue which is part of a PKA consensus sequence well conserved among eukaryotes (RKPS_109_LVA). This serine is dephosphorylated within one hour of progesterone stimulation or in response to experimental PKA inhibition using PKI [43]. Importantly, a phosphomimetic S109 mutant, S109D-ARPP19, proved to be a strong inhibitor of Cdk1 activation when injected into oocytes, not only in response to progesterone but also following PKI, Mos or Cyclin B injections [43]. PKA-dependent phosphorylation of Arpp19 at S109 thus restrains Cdk1 activation and is sufficient to explain the oocyte prophase-arrest in *Xenopus*. Upon progesterone stimulation, PKA is downregulated and Arpp19 is dephosphorylated at S109, a necessary event to initiate the signalling cascade that ultimately leads to MPF activation [43]. The critical molecular interacting partner(s) of phospho-S109- versus dephospho-Arpp19 in the oocyte cytoplasm responsible for this important switch remain to be discovered.

For Arpp19 to act as a critical part of the oocyte maturation initiation machinery requires a specific phosphatase to dephosphorylate serine 109. This phosphatase was recently identified as a specific isoform of the Serine/Threonine protein phosphatase PP2A, PP2A-B55δ [177]. PP2A-B55δ is already active in prophase-arrested oocytes, but its action on Arpp19 is overwhelmed by that of PKA. When PKA activity decreases in response to progesterone, PP2A-B55δ remains active [177], such that Arpp19 becomes dephosphorylated at S109. The prophase arrest of the *Xenopus* fully-grown oocyte is thus governed by a highly dynamic competition between PKA and PP2A-B55δ, which are engaged in a futile cycle of phosphorylation/dephosphorylation of a common substrate, Arpp19. This futile cycle presumably allows the two opposed active enzymes to carry other important functions independently of each other. The discovery of PP2A-B55δ as the enzyme responsible for the dephosphorylation of Arpp19 on S109 was a surprise. Cdk1 activation takes place 3 to 5 h after the dephosphorylation of Arpp19 at its PKA site by PP2A-B55δ. At that time, Cdk1 activation requires this same PP2A-B55δ isoform to be inhibited, and this inhibition is achieved by Arpp19 [114,178]! Indeed, Arpp19 is transformed into a specific inhibitor of PP2A-B55δ when phosphorylated at a distinct Serine, S67, by Gwl [179,180]. Arpp19 and PP2A-B55δ are therefore a central duo of meiosis resumption, assuming two distinct functions during two different critical periods: Arpp19 phosphorylation at S109 by PKA maintains the prophase arrest. Upon hormonal stimulation, Arpp19 is dephosphorylated at S109 by PP2A-B55δ to launch a signalling cascade [43,177]. At the end of the cascade, Arpp19 is phosphorylated at S67 by Gwl, inhibits PP2A-B55δ, becoming a positive actor of Cdk1 activation. Hence, the progression of meiosis maturation relies on the temporal coordination of Arpp19 phosphorylations, achieved by one single phosphatase, PP2A-B55δ, opposing two kinases, first PKA and then Gwl.

### 3.2. Clytia

The identification of MIH in several hydrozoan species as WPRPamide and related tetrapeptides [37] opened the path to the recent discovery of the *Clytia* MIH receptor (MIHR). This GPCR was selected from candidate receptors identified from the oocyte using a “deorphanisation” approach involving their expression in mammalian cultured cells along with a Ca^++^-Aequorin based reporter system [38]. The in vivo function of *Clytia* MIHR was confirmed by CRISPR-Cas9-mediated mutation of the endogenous gene. Female jellyfish carrying MIHR mutations failed to undergo oocyte maturation or spawning in response to the physiological dark-light cue, such that fully-grown oocytes accumulated in swollen gonads. A second phenotype observed in some MIHR mutants, characterised by poor gonad growth, may relate to a distinct role for the MIH-MIHR signalling system in the regulation of nutritional balance in the jellyfish.

Activation of the *Clytia* MIHR by MIH peptides results in the initiation of oocyte maturation, almost certainly via the association of this receptor with Gα_S_ [38]. Receptor activation would release Gα_S_ to activate adenylate cyclase, thereby causing cAMP levels to rise. Consistently, a rise in cytoplasmic cAMP concentration has been demonstrated using oocytes of another hydrozoan, *Cytaeis*, occurring within 2 min of the physiological light-dark cue that induces maturation in this species [58]. Furthermore, in *Cytaeis* and *Clytia* as in other hydrozoans, oocyte maturation can be induced using cell-permeable cAMP analogues [58,181]. Isolated gonads or isolated fully-grown oocytes from MIHR CRISPR mutant *Clytia* jellyfish could also be induced to mature by treatment with cAMP analogues, consistent with cAMP acting downstream of MIHR. Evidence that Gα_S_ links MIHR activation to the cAMP rise was provided by the injection of wild type oocytes with an inhibitory antibody specific to this α_S_ subunit. Anti-Gα_S_ -injected oocytes responded only poorly to endogenous (light-released) or synthetic MIH peptides [38]. Taken together, these results strongly suggest that the *Clytia* MIHR acts through Gα_S_ to stimulate adenylate cyclase and thus causes the cAMP concentration to rise in the cytoplasm. This cAMP signal is likely mediated by PKA, since pharmacological PKA inhibitors prevent maturation of *Cytaeis* eggs [58], implying that in hydrozoans one or more PKA substrates are involved in activating MPF and thus driving entry into first M-phase. The identity of these substrates is not known, however, given that GVBD occurs within 10–15 min of MIH stimulation, it is likely to act directly to trigger MPF auto-amplification.

Intriguingly, both *Xenopus* and *Clytia* oocytes thus deploy during the MIH response Gα_S_-coupled GPCRs that signal through cAMP: constitutive activity of GRP185 holds the prophase block of *Xenopus* oocytes, while *Clytia* MIHR activation, upon ligand binding, releases that block. This curious situation is discussed further in Section 5.

## 4. Transitioning into the Meiotic Divisions: MPF & MAPkinase

In oocytes from all species, MIHs serve to release the prophase block and stimulate the oocyte to enter into M-phase through MPF activation, but they do so through widely different molecular pathways. There is thus considerable variation concerning the intermediate steps that culminate with MPF auto-amplification. For instance, the involvement of newly synthesized proteins upstream of MPF activation ranges from complete independence to a requirement for MPF components themselves. On the other hand, synthesis of Mos protein from oocyte mRNAs is required in almost all oocytes. Its role is to gradually increase MAPkinase activity in the oocytes as maturation proceeds, and thus to ensure the correct positioning of the meiotic spindles as well ultimately as CSF arrest.

In regards to the protein synthesis requirement for MPF activation, the first situation is seen in small mammalian rodents (mouse, rat) and many invertebrate species, where MPF activation and its first cellular consequence, the breakdown of the nuclear envelope (GVBD), do not require protein synthesis. Hence, MIHs promote the conversion of the inactive pre-MPF stockpile into active MPF through a rapid (10 min to 1 h, according to species) cascade of phosphorylations ending with Cdc25 activation and Myt1/Wee1 inactivation, and, as a consequence, Cdk1–Cyclin B activation. Although the synthesis of neither Mos kinase nor Cyclin B is required for initial Cdk1 activation in these species, these newly synthesized proteins subsequently ensure the successful transition from meiosis I to meiosis II by controlling the formation of functional microtubular spindles and by preventing DNA synthesis between the two meiotic divisions, as well as parthenogenetic activation [182,183,184].

A second situation is observed in a variety of fish and amphibian species whose oocytes are devoid of Cyclin B, and consequently of inactive pre-MPF. This is the case in goldfish [185,186,187], catfish [188], zebrafish [188,189,190], the newt *Cynops* [191] or the frog *Rana japonica* [185]. In these species Cyclin B thus has to be synthesized de novo in response to hormonal stimulation to generate active MPF [192].

The third situation covers many vertebrates, including fish and amphibian species as well as nearly all mammals examined except small rodents [193,194,195,196,197,198,199]. In oocytes from these animals, new proteins must be synthesized to initiate MPF activation, despite the presence of a stockpile of inactive Cdk1-Cyclin B. A lot of studies have been dedicated to the identification of these newly synthesized proteins and have focused on two main candidates: Mos and Cyclin B. Synthesis of either of these two proteins is sufficient to promote meiosis resumption and MPF activation in *Xenopu*s oocyte [200,201]. Mos activates a MAPkinase kinase, MEK, which in turn activates MAPkinase, influencing cytoskeletal dynamics during the meiotic divisions, resulting finally in the activation of the CSF component p90^Rsk^ [33]. In oocytes of many species, the MAPkinase activation system appears to operate largely independently of MPF activation during MI entry. In *Xenopus*, however, interfering with Mos synthesis strongly delays Cdk1 activation and GVBD induced by progesterone, although it does not prevent it [202]. At later stages of meiotic maturation in *Xenopus* as in other animal oocytes, Mos is required after meiosis I to prevent DNA synthesis and to arrest oocyte meiosis until fertilisation [202]. Another event important for priming MPF activation in *Xenopus* is the synthesis of Cyclin B. In response to progesterone, Cyclin B1, which is expressed at very low levels in comparison to Cyclin B2 in the prophase-arrested oocyte, is synthesized upstream of Cdk1 activation [105,198]. It associates with monomeric free molecules of Cdk1, forming a small pool of active MPF that can bring about Cdc25 activation and Myt1 inactivation, thereby establishing an auto-activation loop between the new active MPF complexes and the pool of existing inactive pre-MPF through the phosphorylations of Cdc25 and Myt1 [107,108]. However, the destruction of all Cyclin B mRNAs by an antisense approach did not block MPF activation in response to progesterone [203]. In contrast, preventing synthesis of both Mos and Cyclin Bs completely did block MPF activation in response to progesterone [49], indicating that each individual pathway is dispensable, provided that the other one remains functional. The most probable scenario is that newly assembled Cdk1-Cyclin B1 escapes inhibition by Myt1, and thus triggers the initial activation of pre-existing Cdk1-Cyclin B2 through inactivating phosphorylations of Myt1 [49,204]. The Mos–MAPkinase pathway then participates by contributing to activating phosphorylations of Cdc25. Thereafter, a much larger population of pre-existing Cdk1-Cyclin B2 is activated through the auto-regulatory activation loop.

The molecular link from progesterone to de novo synthesis of Mos and Cyclin B1 is still unknown. Does Arpp19 somehow repress Mos and/or Cyclin B synthesis when phosphorylated by PKA? Or does it promote their translation when dephosphorylated by PP2A-B55δ in response to progesterone?

In contrast with the diversity of signals and signalling pathways converging on an initial step of MPF activation, the autoregulatory loop that allows MPF to amplify its activity is a well-conserved process across species. This key circuit controls the abruptness and irreversibility of entry into both mitotic and meiotic division. The basic scheme of this loop is the following. The new complexes formed by the association of synthesized Cyclin B1 and Cdk1 are directly active and bring about some level of Cdc25 activation and Myt1 inactivation. The reversal of this Cdc25/Myt1 balance leads to the activation of some pre-MPF molecules that fire the loop. The more Cdk1 is activated, the more it activates its own activator, Cdc25, and inhibits its own inhibitor, Myt1, and the more it activates itself. Importantly, this switch-like transition is counterbalanced by PP2A-B55δ activity. This phosphatase opposes Cdk1 activity by dephosphorylating Cdc25 and Myt1, thus compromising the auto-amplification process [114,205,206]. This negative effect is avoided by the implication of the kinase Gwl and its substrate, Arpp19. By activating Gwl, Cdk1 enables Arpp19 phosphorylation at S67. Phosphorylated at S67, Arpp19 is converted into a strong inhibitor of PP2A-B55δ [178,179,180,207]. Consequently, the phosphatase counteracting Cdk1 is inactivated and the irreversible switch that engages the cell to divide is turned on.

## 5. Conclusions and Perspectives

The comparison presented here of the progression of oocytes through successive phases of the prophase arrest in *Xenopus* and *Clytia* raises a number of interesting issues. We will conclude this review by identifying some of the shared features as well as the differences, extending where possible the comparison to other animal models. Unfortunately, detailed information about oocyte maturation mechanisms is available only from a scattered sampling of animals, so a rigorous phylogenetic mapping of characters to trace their evolutionary history is not possible, but some lessons can still be drawn.

Firstly, we can consider common features. Like all other meiotic cells, oocytes make a very particular detour from the cell cycle programme when they transition from undergoing mitotic divisions to meiotic divisions. They pause during the preceding S-phase and then during the chromosome pairing, synaptonemal complex formation and crossing over; for instance, in *Xenopus* it takes 7 days for S-phase, 4 days for leptotene, 5 days for zygotene, 18 days for pachytene [208]. There is little information available at present from animal oocyte models about how prophase is initially put on hold during these events, but these are certainly evolutionarily ancient and most probably involve conserved checkpoint mechanisms that monitor DNA breaks [59,60]. Oocyte growth itself also involves some common features across species, albeit deployed in different combinations. Cell-autonomous cell growth programmes involving massive mRNA, protein and organelle synthesis are regulated at least in part by InsR-TOR signalling. This is supplemented by the incorporation of components produced in distant organs and/or by neighbouring cells. One notable difference is that yolk vesicles form by vesicle uptake in *Xenopus* but mainly from the oocyte Golgi in *Clytia*.

Ongoing communication between the oocytes and the soma is important in all species. Thus, for instance, the sensitivity of oocytes to MIHs can be affected by other hormones produced by different tissues. This is the case in scallops, where the expression of the serotonin receptor in the gonad is upregulated upon maturity by estradiol produced in ovaries [1,115]. In vertebrates, estradiol produced by ovarian follicle cells controls the production of vitellogenin for the oocyte by the liver. Additional ways to prevent premature ovulation in relation to seasonality include secreted inhibitors, either small peptides or proteins, originating from oocytes or somatic cells in the gonad or the central nervous system, as described in bivalve molluscs [209,210,211] and mammals [212,213]. Follicle cells and other cells in immediate contact with the oocyte are also, of course, important in breaking the prophase arrest though MIH secretion, but also contribute to its maintenance through gap junction communication. In various species, gap junction communication has been found to discontinue once maturation starts, while the experimental isolation of fully-grown oocytes from the follicle can favour spontaneous maturation [39]. Mammals show an extreme case of this, with traffic of cGMP through gap junctions having evolved to take the role of extracellular MIH signalling [5]. In *Xenopus*, gap junctions between follicle cells and oocytes are not required to maintain the prophase arrest of the Stage VI oocyte, but at earlier stages promote endocytosis of the yolk [62,63]. In hydrozoan species, we have seen that extensive cellular contacts of various kinds between the oocytes and connected endodermal or nurse cells, including large cytoplasmic bridges and cell engulfment, are important in fuelling growth. Gap junctional communication has also been demonstrated between the oocyte and overlying ectodermal cells, but its role is not known [41].

The most obvious and best understood commonality in oocyte prophase block regulation relates to the main molecular actors in M-phase entry once maturation commences. The core biochemical machinery centred on MPF that drives all oocytes into first meiotic M-phase is highly conserved between species and indeed is deployed to drive entry into meiotic and mitotic M-phases across all eukaryotic organisms. In this context, it is useful to extend the conception of MPF beyond the Cdk1-Cyclin B complex to include the kinases (Wee1/Myt1) and phosphatase (Cdc25) immediately involved in its auto-amplification, and also the link via Greatwall and Arpp19 to the parallel inhibition of PP2A [214] (Figure 1). The coordinated activations and inactivations of these components abruptly transform the organisation of the cytoskeleton, nucleus and cytoplasm from an interphase to an M-phase state.

Given that this obligatory biochemical module is inherently unstable, there are many potential ways to trigger it. It is thus unsurprising that different species have evolved a range of different triggering mechanisms. As detailed in Section 4 above, even among vertebrate oocytes, MI entry is assured by many mechanisms. In mouse fully-grown oocytes, all the protein machinery for MPF activation is in place and its activation process is entirely post-translational, while oocytes of some amphibia and fish completely lack Cyclin B such that synthesis of this essential MPF component is required first. *Xenopus* shows an intermediate situation, where synthesis of Cyclin B and/or Mos by the oocyte is required to trigger the activation of preformed Cdk1-Cyclin B complexes. This situation has opened the evolutionary door to the involvement of new actors to reinforce the oocyte prophase arrest, such as PKA-phosphorylated Arpp19 in *Xenopus*. At present, we do not know at what level this molecule feeds into the MPF amplification system, but there are several possibilities. A prime candidate is the protein synthesis machinery itself, since M-phase entry cannot proceed without neosynthesis of either Cyclin B or Mos. Other potential targets are the variety of other kinases and phosphatases with regulatory roles in the cell cycle, which have become specialised during evolution to allow the MPF activation cassette to be blocked or activated in cell-type specific ways. Thus, Wee1 phosphorylation by PKA in mouse oocytes reinforces its inhibitory activity [48].

Vertebrate oocytes are not typical in their requirement for a cAMP concentration decrease for MIH signalling. Rather, for the oocytes of some ascidians, echinoderms, annelids, nemerteans and cnidarians, positive cAMP signals actively trigger entry into meiotic division [39]. Based on the phylogenetic distribution of the various species investigated, it is tempting to speculate that cAMP played an activatory role in oocyte maturation in the earliest metazoans, and that the mechanism for physiological regulation upstream of MPF activation has undergone modifications during evolution of the vertebrate lineage. This may have included the co-option of Arpp19 for an inhibitory role, under the control of PKA. Under this scenario, the ancestral role of Arpp19 was likely the second one observed during *Xenopus* oocyte maturation, namely its highly conserved M-phase role, i.e., in enhancing MPF activation when phosphorylated by Gwl [215]. To explore this hypothesis, it will be of interest to investigate whether *Clytia* Arpp19 is a PKA substrate, and how it interacts with the MPF activation machinery.

The features that are completely divergent between the *Xenopus* and *Clytia* prophase arrested oocyte centre on the MIH signals and their receptors. In one case, the signal is provided mainly by steroids. These may well act via more than one type of receptor and through partly redundant pathways to kick off adenylate cyclase inhibition and a drop in the cytoplasmic concentration of cAMP. In the other, a peptide hormone acts on a GPCR coupled to Gα_S_ to produce a positive cAMP signal.

Some clues to understanding this paradox come from comparing the involvement of GPCRs, which are a common feature of oocyte maturation regulation across species [216]. *Clytia* MIHR is one of very few MIH receptors identified conclusively at the molecular level in any species. We can confidently place it as the physiological initiator of oocyte maturation, linking light-triggered MIH release from the gonad ectoderm, via Gα_S_ and adenylate cyclase, to cAMP elevation and PKA activation in the oocyte. MIH-related peptides and cAMP analogues can trigger oocyte maturation across a range of hydrozoan species [37,181], so this mechanism is likely shared at least in a large group of cnidarian species. GPCRs have also been implicated in triggering the maturation of mollusc and annelid in response to serotonin (5-HT) [1,32]. A diversity of serotonin receptors and downstream pathways appears to be deployed across species [1,217]. These serotonin receptors are in a different GPCR superfamily from *Clytia* MIHR and have wider roles in reproductive regulation, with their role in maturation initiation likely redundant with other pathways, as shown in the nemertean *Cerebratulus* [36]. One of the best characterised to date is a serotonin receptor coupled to Gα_i_ in scallop oocytes, thought to signal via IP3/DAG and the activation of membrane voltage-dependent Ca^2+^ channels.

Intriguingly, vertebrate oocytes possess active Gα_S_-coupled GPCRs that signal through cAMP (GRP185 in *Xenopus* [116,117], GRP3 in mouse and human [118,218], GPR12 in rat [119]). Rather than releasing the prophase arrest as *Clytia* MIHR does, the role of these GPCRs is to sustain the prophase meiotic arrest by constitutive activity that maintains increasing intracellular levels of cAMP via Gα_S_, as well as by Gβγ signalling. Similarly, GPCR30 (GPER-1), a GPCR from another distinct superfamily which also likely signals through both Gα_S_ and Gβγ, participates in maintaining the prophase arrest in fish oocytes through its function as an estrogen receptor [219]. These GPCRs are possible targets of one serotonin receptor antagonist that induces the maturation of vertebrate oocytes [220].

An unidentified GPCR is also involved in mediating the maturation response of starfish oocytes. In response to binding of the MIH 1-methyl-adenine, it promotes the dissociation of Gβγ from Gα_i_ [40,41,221]. In this case, Gβγ is the key signalling intermediate, the critical pathway to MPF activation passing via phosphatidyl-inositol 3,4,5-triphosphate (PIP3) production, with the kinases Akt and especially SGK finally phosphorylating and activating Myt1 and Cdc25 to initiate MPF autoactivation [222,223]. In ascidians, a vasopressin-like peptide has recently been shown to act as an MIH and is likely to signal via a GPCR expressed in late growth stage oocytes, possibly via MAPkinase signalling [224].

Whether the progestin receptors (mPRs) in fish and amphibia are GPCRs has been a matter of debate. The recent consensus is that these mPRs are not classic GPCRs, but belong to a distinct class of membrane receptors spanning seven (or eight) times the plasma membrane, called PAQRs (progestin and adipoQ receptors), which include receptors for adiponectin in vertebrates [219,225]. PAQRs have a different topology, motif structure and ancestral origin to classic GPCRs [219,226,227]. Nevertheless, several studies have shown that mPRs (mPRα and mPRβ) in the membranes of fish [29,141,226,228,229,230,231] and *Xenopus* [101] oocytes are specific progestin receptors that activate G proteins and thus act functionally in the same way as GPCRs. Studies on fish oocytes showed that the inhibition of cAMP production by mPRα is blocked by pertussis toxin, suggesting the involvement of an inhibitory G_i_ protein [29,141,230], whereas *Xenopus* mPRβ would act through a pertussis toxin-insensitive pathway [101]. Hence, the activities of these receptors have been described as mediating progestin-induced meiotic maturation via G-proteins and subsequent lowering of cAMP levels and PKA activity in several fish species and also, by a different pathway, in *Xenopus*. One way that mPRs could signal independently of G-proteins is through sphingolipids, since PAQR family receptors have sequence motifs characteristic of alkaline ceramidases and corresponding enzymatic activity [232,233]. The ceramidase activity of mPRβ to generate sphingosine 1-phosphate could potentially modulate the endocytosis of the constitutively active GPR185 in *Xenopus* [126], as has been shown for the equivalent receptor (GPR3) in mammalian oocytes [126,234,235]. It is also possible to envisage a dual mechanism for mPR involving both ceramidase stimulated endocytosis and G protein signalling [227].

Analysis of the evolutionary relationships amongst the different GPCRs involved in oocyte maturation revealed that the *Clytia* MIHR is not closely related to GRP185/GRP3 or to the serotonin receptors. Amphibian and fish mPRs, as discussed above, belong to a distinct family from classic GPCRs. Within this PAQR family, the mPRs first appeared early in vertebrate evolution, before the divergence between teleosts and tetrapod lineages [226]. *Clytia* MIHR belongs to a family of neuropeptide hormone receptors including the receptors for vertebrate Neuropeptide Y, QRFP and GnIH [38]. These all act at the level of the hypothalamus/pituitary and, amongst other functions, allow GnRH and gonadotropin release at those sites to be modulated in relation to stress, nutrition and other physiological parameters. It is thus tempting to speculate that a GPCR of this particular neuropeptide hormone receptor superfamily already had a role in regulating gamete production in a distant animal ancestor. Subsequent complexification of the reproductive regulation, including the introduction of specialised tissues such as follicle cells and endocrine organs and parallel expansion of the receptor and ligand families, would have resulted in the scattered involvement of these GPCRs today at different levels of spanning regulation, including as MIH receptors in hydrozoans [37,38]. Other hormone systems with widespread involvement in reproduction including serotonin, estrogen and insulin will have undergone similarly complex evolutionary histories in parallel. Information about their receptors from many more species will be very informative to explore these ideas further.

To conclude, the variety of mechanisms governing the oocyte prophase block between species and also between different phases of oogenesis at first seems bewildering, but valuable lessons can emerge from the comparative approach. Separating the prophase block into successive phases is also useful for making sense of the molecular complexity. Conserved mechanisms govern the initial programme of the nascent oocyte as it completes synapsis prior to oocyte growth and also, much later, the entry into an M-phase state once the block has been released. In contrast, the maintenance of a prophase state once the oocyte has reached full size depends on highly species-specific mechanisms, which are progressively put in place during growth. The observed confusion of hormones, ligands and receptors and downstream pathways reflects the complex evolutionary history of sexual reproductive regulation. More specifically, frequent acquisition in different animal evolutionary lineages of new MIH-MIHR systems may reflect contradictory pressures to generate good quality gametes and to respond rapidly and reliably to hormonally-guided ovulation cues. One consequence of the oocyte’s complex evolutionary history is that multiple signalling pathway components are available in the cytoplasm to converge on MPF activation. The resultant functional redundancies in the maturation initiation response, for instance between Mos and Cyclin B synthesis in *Xenopus* oocytes, create headaches for the researcher but add robustness to the systems to ensure a reliable outcome to the vital process of generating a successful gamete.

Finally, by highlighting the value of a comparative approach, this review makes clear that our understanding of the regulation of oocyte maturation would greatly benefit from more in-depth knowledge from a wider range of species. In this context, the current drive to develop new experimental model species for cell and developmental biology should certainly be encouraged.
